# New Insights into the Role of Insulin and Hypothalamic-Pituitary-Adrenal (HPA) Axis in the Metabolic Syndrome

**DOI:** 10.3390/ijms23158178

**Published:** 2022-07-25

**Authors:** Joseph A. M. J. L. Janssen

**Affiliations:** Department of Internal Medicine, Erasmus Medical Center, Room Rg527, 3015 GD Rotterdam, The Netherlands; j.a.m.j.l.janssen@erasmusmc.nl; Tel.: +31-06-50032421 or +31-10-7040704

**Keywords:** hyperinsulinemia, relative hypoinsulinemia, insulin, insulin resistance, insulin receptor, westernized diet, over-nutrition, metabolic syndrome, Cushing syndrome, cortisoluria, CBG, 11β-HSD1

## Abstract

Recent data suggests that (pre)diabetes onset is preceded by a period of hyperinsulinemia. Consumption of the “modern” Western diet, over-nutrition, genetic background, decreased hepatic insulin clearance, and fetal/metabolic programming may increase insulin secretion, thereby causing chronic hyperinsulinemia. Hyperinsulinemia is an important etiological factor in the development of metabolic syndrome, type 2 diabetes, cardiovascular disease, polycystic ovarian syndrome, and Alzheimer’s disease. Recent data suggests that the onset of prediabetes and diabetes are preceded by a variable period of hyperinsulinemia. Emerging data suggest that chromic hyperinsulinemia is also a driving force for increased activation of the hypothalamic-adrenal-pituitary (HPA) axis in subjects with the metabolic syndrome, leading to a state of “functional hypercortisolism”. This “functional hypercortisolism” by antagonizing insulin actions may prevent hypoglycemia. It also disturbs energy balance by shifting energy fluxes away from muscles toward abdominal fat stores. Synergistic effects of hyperinsulinemia and “functional hypercortisolism” promote abdominal visceral obesity and insulin resistance which are core pathophysiological components of the metabolic syndrome. It is hypothesized that hyperinsulinemia-induced increased activation of the HPA axis plays an important etiological role in the development of the metabolic syndrome and its consequences. Numerous studies have demonstrated reversibility of hyperinsulinemia with lifestyle, surgical, and pharmaceutical-based therapies. Longitudinal studies should be performed to investigate whether strategies that reduce hyperinsulinemia at an early stage are successfully in preventing increased activation of the HPA axis and the metabolic syndrome.

## 1. Introduction

In the Western populations there is a cluster of metabolic risk factors which include hypertension, glucose intolerance, abdominal obesity, and hyperlipidemia. Since these factors are observed together more frequently than by chance alone, the concept was developed that these factors are interrelated and together produce the metabolic syndrome [1]. The metabolic syndrome increases risks for developing cardiovascular diseases (such as heart attacks and strokes), type 2 diabetes mellitus, and cancer. The metabolic syndrome has its own set of underlying risk factors of which hyperinsulinemia and insulin resistance, defined as a subnormal response (of blood glucose levels) to insulin, are considered the most important. Other risk factors for the metabolic syndrome are abdominal adiposity, physical inactivity, aging, stress, sleep disturbance, circadian disruption, and the Western diet.

For many years, the dogma has been that insulin resistance was the primary etiological factor in the development of obesity, the metabolic syndrome, and type 2 diabetes, and preceded hyperinsulinemia [2,3]: hyperinsulinemia was considered secondary representing a compensatory mechanism to overcome systemic (peripheral) insulin resistance. However, at present there is no satisfactory explanation for how insulin resistance might stimulate insulin secretion, while there are only a few naturally occurring or genetic models of primary insulin resistance, and few diabetes genes are implicated in insulin resistance [3].

**Figure 1:**
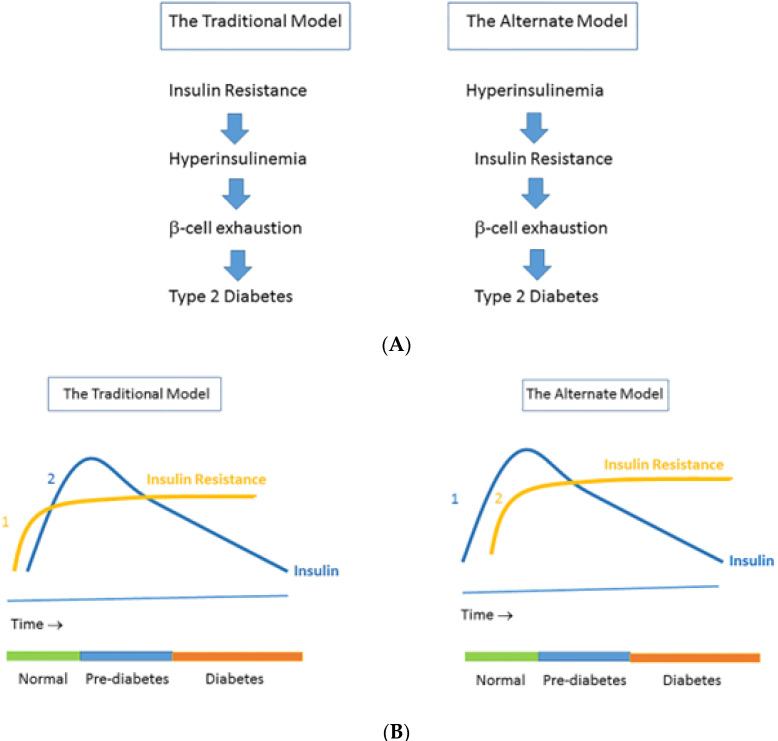
(**A**) The traditional model vs. the alternate model of the pathogenesis of insulin resistance. The traditional model (which is a widely held view) posits that insulin resistance leads to hyperinsulinemia, which in time is followed by β-cell dysfunction (**left**). In the traditional model, insulin resistance (1) precedes hyperinsulinemia (2), which may be followed by β-cell exhaustion and finally frank type 2 diabetes. (**B**) In the alternate model, hypersecretion of insulin and the resulting hyperinsulinemia (1) primarily cause insulin resistance (2), which may be followed by β-cell exhaustion and finally frank type 2 diabetes (**right**). Note that in the alternate model hyperinsulinemia is already present when there is still a normal glucose tolerance. Reproduced from [12].

**Figure 2:**
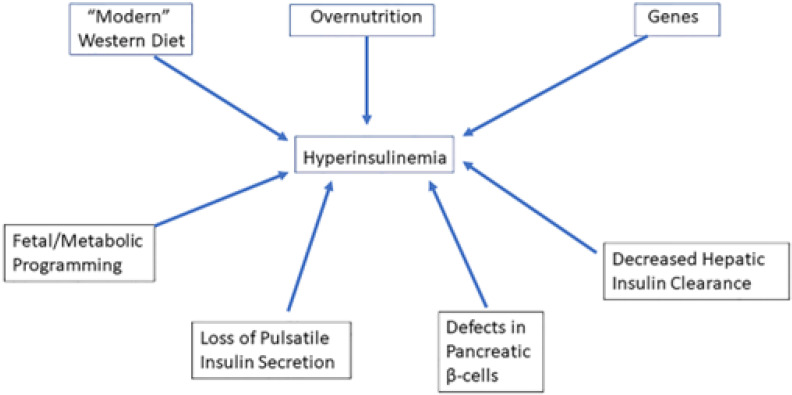
Factors involved in primary hyperinsulinemia. Consumption of the “modern” Western diet, over-nutrition, genes, defects in pancreatic β-cells, decreased hepatic insulin clearance, loss of pulsatile insulin secretion, and fetal/metabolic programming may increase insulin secretion, thereby causing chronic hyperinsulinemia and hyperinsulinemia-induced insulin resistance. In the alternate concept, hyperinsulinemia precedes impaired insulin secretory function during the conversion from normoglycemia to (pre) diabetes onset: hyperinsulinemia-induced insulin resistance is considered a physiological protective defense mechanism of the body that tries to prevent hypoglycemia and to protect critical tissues from metabolic stress and nutrient-induced injury.

The direct contributions of insulin itself in causing or sustaining insulin resistance have received little sustained attention [2]. Recent data place hyperinsulinemia mechanistically upstream of insulin resistance and suggest that insulin hypersecretion, rather than beta cell dysfunction, identifies otherwise normal individuals at risk for type 2 diabetes [4]. After a period of 3 years, Ferrannini et al. found in a prospective study that subjects with higher insulin secretion at baseline were more likely to progress to impaired glucose tolerance or T2D than those with lower insulin secretion [5]. This result, which is consistent with other human studies [6,7,8,9,10], suggests thus that primary insulin hypersecretion is a triggering event in T2D pathogenesis [11,12] (Figure 1). In this alternate concept, hyperinsulinemia precedes impaired insulin secretory function during the conversion from normoglycemia to (pre)diabetes onset: hyperinsulinemia-induced insulin resistance is considered a physiological protective defense mechanism of the body that tries to prevent hypoglycemia and to protect critical tissues from metabolic stress and nutrient-induced injury [12,13,14]. Thus, hyperinsulinemia may be both a result and a driver of insulin resistance [2]. In this latter case, hyperinsulinemia may be the direct consequence of consumption of the “modern” Western diet, overnutrition, decreased hepatic insulin clearance, genetic factors, fetal/metabolic programming, defects in pancreatic β-cells, and loss of pulsatile insulin secretion [12] (Figure 2).

The metabolic syndrome and Cushing syndrome share several features, including abdominal visceral obesity, insulin resistance, impaired glucose tolerance, and type 2 diabetes mellitus, hypertension, and hypertriglyceridemia (Table 1). The overlap in clinical characteristics between the metabolic syndrome and Cushing syndrome may be caused by common underlying mechanisms. Earlier studies did not see consistent relationships between plasma cortisol levels and the presence of the metabolic syndrome [15]. However, new data suggest (a mild) hyperactivity of the HPA axis inducing a state of “functional hypercortisolism” in subjects with the metabolic syndrome [16,17,18]. Despite the hyperactivity of the HPA axis, in most subjects with the metabolic syndrome, plasma cortisol concentrations are usually low/normal, while simultaneously urinary free cortisol clearance is increased [16,17,18] (Table 1). It has been suggested that increased cortisol clearance is responsible for the observed low plasma cortisol concentrations in subjects with the metabolic syndrome. With decreased negative feedback at the hypothalamus and the pituitary, low plasma cortisol might in turn result in an enhanced adrenocorticotropic hormone (ACTH)-induced cortisol response [19]. An alternative explanation for the low/normal plasma cortisol concentrations in subjects with the metabolic syndrome might be hyperreactivity of the target cells to cortisol [20].

Subjects with endogenous Cushing syndrome and the metabolic syndrome differ significantly with respect to insulin secretion. While hyperinsulinemia in the metabolic syndrome is primary, hyperinsulinemia in endogenous Cushing syndrome is secondary, representing a compensatory mechanism to overcome insulin resistance. Moreover, while hyperinsulinemia is an obligatory finding in subjects with the metabolic syndrome, subjects with endogenous hypercortisolemia (Cushing syndrome) and impaired glucose tolerance show a relative hypoinsulinemia, wherein insulin levels have increased, but less than would be expected for the level of plasma glucose [21]. In subjects with Cushing syndrome as the endogenous hypercortisolemia exacerbates, the relative insulinopenia becomes more paramount, suggesting that relative insulinopenia is caused by cortisol-mediated direct or indirect “toxic” effects on the pancreatic β-cells, which suppresses endogenous insulin secretion [21,22]. As a direct consequence, insulin secretion in subjects with Cushing syndrome will not suffice to adequately control plasma glucose in response to a glucose load [21,22,23] (Table 1). Excess cortisol has negative effects on insulin secretion, insulin sensitivity, and glucose tolerance but does not primarily induce hyperinsulinemia (see below paragraph *Effects of glucocorticoids on insulin secretion* for more details).

In this paper, data are presented showing that the hyperinsulinemia-mediated increased activation of the HPA axis may play an etiological role in the development of the metabolic syndrome. It is postulated that hyperinsulinemia-induced “functional hypercortisolism” disturbs the energy homeostasis of the body and functions as a physiological defense mechanism, preventing hypoglycemia and protecting muscles and other critical tissues from metabolic stress and nutrient-induced injury by shifting energy from the muscles to visceral adipocytes [12]. Before going into more detail, background information about the HPA axis and insulin will be first provided in the next paragraphs.

## 2. Regulation of the Hypothalamic-Pituitary-Adrenal (HPA)-Axis in Healthy Subjects

**Figure 3:**
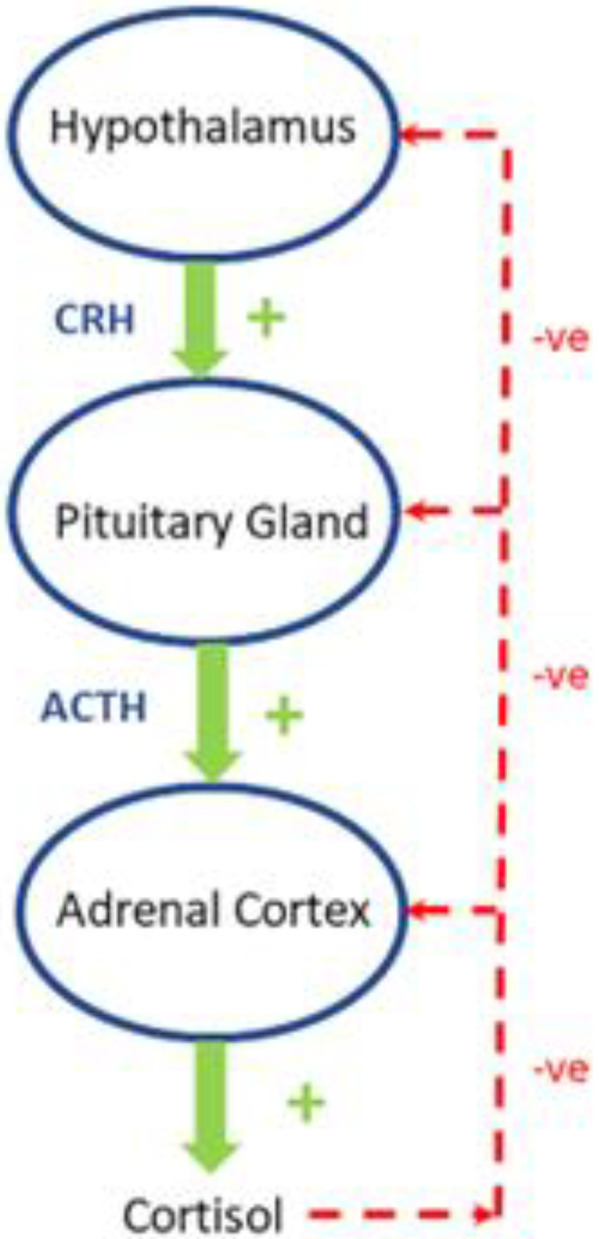
In healthy subjects, corticotropin-releasing hormone (CRH) stimulates the secretion of adrenocorticotropic hormone (ACTH) in the anterior lobe of the pituitary gland. ACTH in turn acts on the adrenal cortex which produces glucocorticoid hormones (mainly cortisol in humans) in response to stimulation by ACTH. Cortisol in turn acts back on the hypothalamus and the pituitary (to suppress CRH and ACTH) production in a negative feedback cycle. Stimulatory effects are shown in green and inhibitory effects are shown in red. See text for more details.

Hypothalamus and pituitary regulate cortisol synthesis and release in healthy subjects (Figure 3). Corticotropin-releasing hormone (CRH) is released by the hypothalamus and stimulates the anterior pituitary to release ACTH. ACTH then acts on the adrenal cortex which promotes the secretion of cortisol from the zona fasciculata. The secretion of cortisol provides a negative feedback loop by inhibiting release of CRH and ACTH from the hypothalamus and anterior pituitary, respectively. The activity of 11 beta-hydroxysteroid dehydrogenase (11b-HSD) plays an important role in extra-adrenal cortisol metabolism [24]. At least two isozymes of 11 beta-HSD exist, which catalyze the interconversion of hormonally active glucocorticoids (cortisol, corticosterone) and their inactive metabolites (cortisone, 11-dehydrocorticosterone) [25] 11β-hydroxysteroid dehydrogenase-2 (11β-HSD2), is mainly expressed in the kidneys and protects the mineralocorticoid receptor from glucocorticoid excess by converting cortisol to cortisone [25]. It thereby promotes the access of aldosterone to the mineralocorticoid receptors in the kidney. The other isoform, 11β-hydroxysteroid dehydrogenase-1 (11β-HSD1), is widely expressed in classic insulin target tissues as the liver, muscle, and adipose tissue.

Circulating levels of cortisol are regulated through a balance between synthesis in the adrenal cortex and clearance via metabolic pathways in the liver. An increase in the level of circulating cortisol may be related to stimulation of 11β-HSD type 1 in the liver and/or reduced cortisol clearance by inhibition of 5α- reductase or 5β-reductase [24].

Cortisol metabolism is not only regulated centrally, but is also regulated peripherally. A rise of 11β-HSD1 activity (locally) at the tissue level stimulates conversion of cortisone to cortisol, and in this way cortisol receptors are locally exposed to increased cortisol concentrations [26,27]. Cortisol itself stimulates 11β-HSD1 expression in hepatocytes, adipocytes, and myoblasts [18]. Moreover, other factors which may increase 11β-HSD1 expression in a tissue-specific manner are tumor necrosis factor α (TNFα), interleukin-1β (IL-1β), and interleukin-6 (IL-6), whereas insulin, insulin-like growth factor-I (IGF-I), and growth hormone reduce 11β-HSD1 activity [18].

Visceral and omental adipocytes show a higher number of cortisol receptors than subcutaneous adipocytes [28]. In addition, 11β-HSD1 in visceral and omental adipocytes converts locally more cortisone to (active) cortisol than subcutaneous adipocytes [29]. As an immediate result, visceral and omental adipocytes are exposed to higher cortisol concentrations than are present in the circulation and this may contribute to the preferential deposition of fat at intra-abdominal rather than subcutaneous sites [28,30].

## 3. How Does Cortisol Affect Intermediary Metabolism?

Cortisol is one of the main hormones that regulate fuel homeostasis. After uptake of free cortisol from the circulation, cortisol exerts its effects by binding to intracellular cortisol receptors [31]. Cortisol increases availability of potential fuel substrates by mobilization of glucose, free fatty acids, and amino acids from the body energy stores (adipose tissue, muscle protein, and glycogen) and thereby stimulates energy expenditure [32].

The peripheral effects of cortisol (i.e., outside the central nervous system (CNS)) are primarily catabolic [9]. Cortisol enhances hepatic gluconeogenesis, inhibits glucose uptake in muscles, and increases circulating free fatty acid levels by increasing adipose tissue lipolysis [33].

Under experimental conditions, the amplitude of the diurnal variations in insulin was significantly related to amplitude of cortisol rhythm, suggesting that cortisol day rhythm may mediate, at least partially, the diurnal variation in carbohydrate tolerance [34]. It has been suggested that cortisol induces increased insulin resistance and in this way may contribute to development of impaired glucose tolerance and the metabolic syndrome [35].

Control of food intake is complex and involves numerous brain neurotransmitters and central and peripheral neural structures. Centrally, the HPA axis is believed to interact with the hypothalamic neurotransmitters mediating effects on nutrient intake [36]. In particular, central effects mediated by CRH and cortisol are primarily anabolic and increase appetite and caloric and food intake [36,37,38]. Elevated cortisol in situations of chronic stress increases appetite and intake of especially nutrient dense food and this makes it possible to quickly replace nutrients that are lost during a fight or flight acute stress response [39].

## 4. How Does Insulin Affect Intermediary Metabolism?

Insulin is considered the master regulator of metabolism in the body [40]. Peripheral actions of insulin (i.e., outside the CNS) are primarily anabolic and stimulate energy storage [29]. In the liver, insulin stimulates glucose utilization, glycolysis, and glycogenesis, and it downregulates glucose production by suppressing gluconeogenesis and glycogenolysis [41]. In the skeletal muscles, insulin facilitates uptake of glucose and amino acids from the bloodstream [42]. Subsequently, glucose is mostly utilized in glycolysis in order to produce energy in the form of ATP which is thereafter used for functional protein synthesis [42]. Insulin converts glucose in muscles to glycogen and further stimulates lipogenesis by promoting uptake of fatty acids into adipocytes. These fatty acids are subsequently converted into triglycerides, which function as long-term energy sources [42]. Simultaneously, insulin inhibits protein degradation (proteolysis) and suppresses lipolysis and ketogenesis.

The circumventricular organs (which include the median eminence and adjacent neurohypophysis) are structures that permit substances that do not cross the blood brain barrier to trigger changes in brain function [43]. Uptake of plasma insulin into the central nervous system (CNS) is mediated by a saturable mechanism consistent with insulin binding to blood-brain barrier and/or blood-cerebrospinal fluid (CSF) barrier insulin receptors and subsequent transcytosis into the CNS [44]. This transport process may be subject to regulation by endogenous factors and interventions that reduce efficiency of insulin uptake in in the CNS [45]. Because the bulk of brain glucose uptake is not affected by insulin in humans, the brain had long been considered “insulin insensitive” [46,47,48]. While there is evidence for glucose transport with the insulin-sensitive GLUT4 in a few selected nuclei in the brain (see below), glucose transport into most neurons is GLUT3-dependent, while the glia and brain endothelial cells depend on GLUT1 activity for glucose uptake from brain interstitial fluid (ISF) and plasma, respectively [49]. Thus insulin does not play a major role in glucose transport into most brain cells [46]. However, insulin functions as a key afferent signal to the CNS for the control of energy balance [50]. In the CNS, insulin has primarily catabolic effects by inhibiting food intake and thereby energy acquisition. In addition, insulin actions in the hypothalamus increase outflow of the sympathetic nervous system to brown adipose tissue and this increases energy expenditure and production of heat by stimulating fatty acid oxidation [50]. In addition, when insulin is infused into the third cerebral ventricle, this induces a sharp decline of hepatic glucose production (HGP) without any changes in circulating insulin levels or the so called counterregulatory hormones [51]. Central insulin signaling suppresses HGP by activating Kupffer cells in the liver to release interleukin-6 (IL-6). IL-6 in turn acts via phosphorylation of signal transducer and activator of transcription-3 (STAT-3) in the liver leading to the suppression of hepatic glucose production [52,53].

The insulin-regulatable glucose transporter, GLUT-4, is expressed in the hypothalamus [54]. The hypothalamus lays outside the blood brain barrier and is therefore sensitive to circulating insulin [54]. It has been further reported that hyperinsulinemia under normoglycemic conditions increases local glucose utilization significantly in the ventromedial, dorsomedial, and anterior hypothalamic nuclei, suggesting that these nuclei are physiologically activated in response to hyperinsulinemia [55]. It has been further proposed that insulin-stimulated uptake of glucose into areas of the hypothalamus represents a form of metabolic feedback regulation, allowing circulating insulin and glucose (with other mechanisms) to maintain blood glucose homeostasis [54].

## 5. The Bidirectional Interactions between Insulin and the HPA Axis

In healthy subjects, insulin normally shows a reciprocal relationship with cortisol: insulin inhibits food intake while cortisol stimulates food intake [56]. Insulin and cortisol are major antagonistic regulators of energy balance. Effects of cortisol and insulin on food intake may be mediated through regulation of hypothalamic neuropeptide-Y (NPY) synthesis and secretion [56]. In the arcuate nuclei, insulin inhibits and cortisol stimulates the expression of NPY mRNA, which may explain in part the reciprocal actions of insulin and the HPA axis on energy acquisition during the day. It has been further suggested that inhibition of insulin transport across the blood brain barrier by glucocorticoids could be the basis for the enhanced appetite seen with glucocorticoid treatments [57].

## 6. Effects of Glucocorticoids on Insulin Secretion

Healthy subjects in the Japanese general population with serum cortisol levels within the normal range showed a significant negative relationship between serum cortisol levels and insulin secretion [58]. When healthy subjects were divided in three equal tertiles based on serum cortisol levels, subjects in the highest tertile were at greater risk of decreased insulin secretion than subjects in the lowest tertile [58]. Further adjustments for insulin resistance confirmed this negative relationship [58]. Overall, these findings suggest that cortisol levels (even within the normal range) suppress insulin secretion [46].

**Figure 4:**
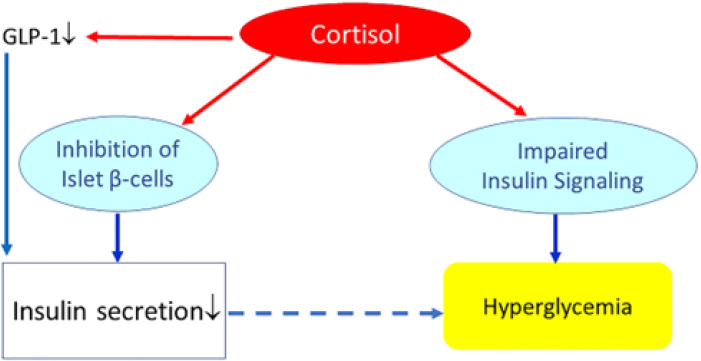
Effects of (high) glucocorticoids on insulin, insulin resistance, and glucose metabolism. Cortisol directly suppresses pancreatic insulin release. It also reduces glucagon-like peptide-1 (GLP-1) production, which further decreases insulin secretion. Increased cortisol also induces glycogenolysis and the expression of key gluconeogenic enzymes, which will increase hepatic glucose production and release. In addition, cortisol may impair insulin receptor signaling (at the receptor and post-receptor level) and thereby induce insulin resistance. Failure of the pancreas to mount an adequate compensatory insulinemic response (“relative hypoinsulinemia” due to cortisol-mediated suppression of pancreatic insulin release) with cortisol-induced insulin resistance may lead to hyperglycemia and impaired glucose tolerance. However, if insulin secretion is sufficient to overcome cortisol-mediated insulin resistance, cortisol will not materially affect glucose tolerance.

Decreased insulin release by pancreatic beta cells is observed following prolonged exposure (days) to glucocorticoids [59,60,61]. Chronic exposure to high circulating glucocorticoid levels inhibits insulin release by binding to glucocorticoid receptors present on pancreatic beta cells [23]. Glucocorticoids reduce the uptake and metabolism of glucose in pancreatic β-cells by genomic actions (i.e., modulation of gene expression by binding of glucocorticoids to the nuclear glucocorticoid receptors). As an immediate consequence, efficacy of cytoplasmic Ca2+ on the exocytosis of insulin granules in the pancreatic β-cells will decrease [59]. Glucocorticoids may further impair pancreatic β-cells glucose metabolism by reducing expression of glucose transporter GLUT-2 and glucokinase thereby decreasing glucose uptake and phosphorylation of pancreatic β-cells [61]. As a direct result, both ATP synthesis and calcium influx, considered essential for a normal pancreatic insulin secretion, decrease and insulin levels after a glucose load will become insufficient to maintain normal plasma glucose levels. Chronic exposure to glucocorticoids may further reduce the insulinotropic effects of glucagon-like peptide-1 (GLP-1) [62]. Thus, with several mechanisms, glucocorticoids may inhibit pancreatic β-cell mediated insulin secretion and thereby induce a “relative hypoinsulinemia”. In addition, glucocorticoids may induce insulin resistance (see next paragraph *Glucocorticoids, insulin resistance and metabolism*). As long as pancreatic insulin secretion is sufficient, cortisol-mediated increase of insulin resistance and hepatic glucose production will not materially affect glucose tolerance. However, failure of the pancreas to mount an adequate compensatory insulinemic response (due to cortisol-mediated suppression of pancreatic insulin release) may lead to hyperglycemia and impaired glucose tolerance (Figure 4).

## 7. Glucocorticoids, Insulin Resistance and Metabolism

Glucocorticoids are associated with insulin resistance and antagonize (counteract) insulin-mediated uptake and utilization of glucose in adipose tissue and muscles [63,64]. These latter effects are mediated by multiple mechanisms: glucocorticoids translocate glucose transporters from the plasma membrane thereby reducing insulin-mediated glucose uptake [64]. In addition, glucocorticoids decrease insulin receptor affinity, induce post-insulin receptor defects by decreasing key mediators of insulin action in peripheral tissues as insulin receptor substrate-1 (IRS-1), phosphatidylkinaseinositol-3 kinase, and protein kinase B [59,65,66]. The liver seems particularly vulnerable to the negative effects of glucocorticoids on insulin actions. In the liver glucocorticoids promote gluconeogenesis by inducing phosphoenolpyruvate carboxykinase [18]. Glucocorticoids further increase gluconeogenesis by potentiating actions of insulin-antagonistic hormones like glucagon [65].

Skeletal muscles play an important role in glucose metabolism. Immediately after a meal, skeletal muscles are responsible for approximately 80% of glucose uptake from the circulation. The skeletal muscles contain the body’s largest glycogen stores. Peripheral skeletal muscle atrophy is a classic sign of prolonged (chronic) exposure to relative high levels of glucocorticoids. When there is peripheral skeletal muscle atrophy due to chronic exposure to relative high levels of glucocorticoids, size of glycogen stores as well as glucose uptake from the circulation will be reduced. In addition, excess glucocorticoids decrease insulin-stimulated glycogen synthase kinase-3 phosphorylation in skeletal muscles which may result in decreased glycogen synthesis [67].

Glucocorticoids further influence insulin sensitivity by modifying lipid metabolism. Adipose tissues from various sites of the body show marked differences in metabolism and sensitivity to glucocorticoids. Glucocorticoids induce hormone sensitive lipase and inhibit tissue lipoprotein lipase activity in peripheral subcutaneous fat cells [68]. This induces an increased fat mobilization from the subcutaneous fat depots. Despite these glucocorticoid-mediated effects on lipolysis, the net effect of glucocorticoid excess is a relocation of fat depots since glucocorticoids simultaneously enhance fat deposition in the visceral compartment [69]. Synergistic effects of glucocorticoids and insulin in visceral adipose tissue induce differentiation and proliferation of fat precursor stem cells and increase tissue lipoprotein lipase [70]. These synergistic effects may contribute to a selective increase of abdominal fat cells and central adiposity, which—as discussed previously—are classical characteristics of both the metabolic syndrome and Cushing syndrome [70]. Thus, contrary to the prevailing opinion that cortisol causes insulin resistance (in all tissues of the body), increased visceral adiposity may be in fact due to synergistic effects of insulin and cortisol on fat cells [35]. Visceral adipose tissue is less sensitive to antilipolytic actions of insulin than subcutaneous adipose tissue. Reduced insulin sensitivity may lead to the release of free fatty acids from visceral fat depots and thereby increase plasma free fatty acid levels. This will further exacerbate insulin resistance and contribute to decreased insulin-mediated glucose uptake and disposal in muscles.

## 8. The Biologically Defended Level of Glycemia in Health and Disease

The biologically defended level of glycemia (BDLG) is determined by the balance between rates of glucose appearance into and disappearance from the circulation, and imbalance in these rates contributes to an elevated BDLG [4]. In health, acute deviations from the BDLG are counteracted by both insulin-dependent and insulin-independent mechanisms that restore blood glucose levels into the normal range [4]. A rise in blood glucose levels stimulate an increased glucose-stimulated insulin secretion, while conversely a fall in blood glucose levels triggers adaptive neuroendocrine and autonomic counterregulatory responses, including an increased HPA activity and cortisol secretion [4]. However, a gradual and pathological increase in the BDLG may occur during the development of type 2 diabetes [4]. As a consequence, the lower boundary of the BDLG is increased and when glucose falls induction of adaptive neuroendocrine and autonomic counterregulatory responses (including an increased HPA activity), occurs already at a higher plasma glucose level. In healthy subjects a rise in plasma cortisol was observed when blood glucose concentrations decreased from 5.5 to 3.5 mmol/L, while in diabetic subjects a rise in plasma cortisol was already observed when plasma glucose decreased from 13 to 5.8 mmol/L [71,72]. It has been further found that the glycemic threshold for triggering counterregulatory responses including the HPA activity (presumably, the level at which the brain perceives a glucose deficit) is elevated by 40% or more in people with T2D compared to nondiabetic individuals [4,73,74]. This may also explain why in routine clinical practice T2D patients frequently report feelings of hypoglycemia when measured plasma glucose levels are within the normal range [73]. Although the underlying mechanism(s) for the increase in glycemic threshold in T2D remains to be elucidated, we hypothesize that hyperinsulinemia may be involved in the gradual and pathological increase in the BDLG during the development of type 2 diabetes (see next paragraphs below for more details).

## 9. Hyperinsulinemia Induces a State of “Functional Hypercortisolism”

As previously discussed, numerous recent data are supportive of the concept that hyperinsulinemia per se is primary and causes insulin resistance [3,11] (Figure 1). In this alternate concept, insulin resistance is proposed to be a physiological defense mechanism of the body preventing hyperinsulinemia-induced hypoglycemia and protecting against overstimulation of target tissues from metabolic stress and nutrient-induced injury [12,13,14]. This concept is even more interesting against data suggesting that hyperinsulinemia per se directly stimulates activation of the HPA axis and thereby induces a state of “functional hypercortisolism”. This “functional hypercortisolism” may help to antagonize insulin actions and in this way prevent both hypoglycemia and overstimulation of target tissues from nutrient-induced injury (see next paragraph entitled *Evidence for insulin regulation of the HPA activity* below for more details).

## 10. Evidence for Insulin Regulation of the HPA Activity

Most people are familiar with the central interactions between insulin and the HPA axis during hypoglycemia. From a physiological perspective, activation of the HPA axis during hyperinsulinemia may develop to prevent an impending fall in blood glucose. Increased release and actions of cortisol after hyperinsulinemia may prevent hypoglycemia by cortisol-mediated effects and enlarging the body’s sensitivity to other counterregulatory hormones [75]. In addition, it was further shown that epinephrine and norepinephrine levels progressively increased with increasing doses of insulin, suggesting that insulin also plays a direct role in stimulating release and effects of catecholamines to prevent hypoglycemia in dogs [76]. The insulin-mediated increase of epinephrine and norepinephrine cause—like cortisol— an elevation of plasma glucose levels in normal humans due to an epinephrine- and norepinephrine-induced suppression of endogenous insulin secretion and a direct inhibitory effect on insulin-stimulated glucose utilization [77].

**Figure 5:**
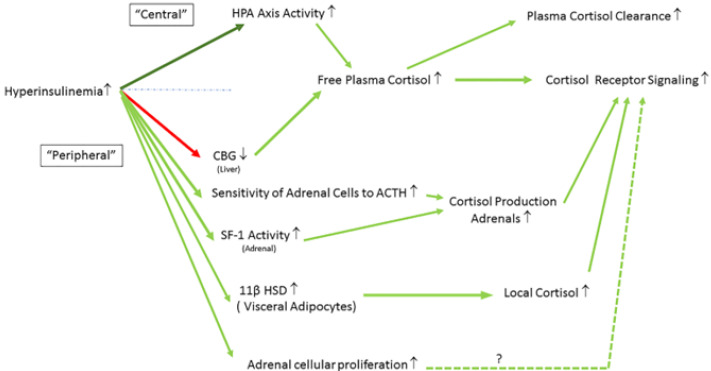
Hyperinsulinemia influences many aspects of cortisol metabolism at both the central and the peripheral level. Interactions are shown between hyperinsulinemia, HPA axis activity, 11β-HSD, sensitivity of adrenals to ACTH, SF-I activity (adrenals), CBG (liver), and adrenal cellular proliferation (See text for more details). Stimulatory effects are shown in green (dark green: central effects; light green: peripheral effects) and inhibitory effects are shown in red. ↑ = increased; ↓ = decreased. List of abbreviations. HPA = hypothalamus-pituitary-adrenal axis activity; CBG = cortisol binding globulin; ACTH = adrenocorticotropic hormone; SF-1 = steroidogenic factor 1; 11β HSD = 11 beta-hydroxysteroid dehydrogenase.

Hyperinsulinemia influences many aspects of cortisol metabolism at both a central and a peripheral level (Figure 5). A rise in plasma insulin during euglycemia (i.e., during normal plasma glucose levels) appears to moderately stimulate activation of the HPA axis in both normal and diabetic animals [78]. During hyperinsulinemic-euglycemic glucose clamps, insulin alone increased HPA activity in normal and diabetic rats by stimulating CRH, vasopressin mRNA, plasma ACTH, and corticosterone [78]. This response was further accompanied by increased glucocorticoid receptor mRNA expression in the anterior pituitary and paraventricular nucleus of the hypothalamus [78]. However, these insulin-mediated effects appeared dose-dependent since the HPA axis was only clearly stimulated during hyperinsulinemia but not when insulin was administered in physiological concentrations [78].

During hyperinsulinemic-euglycemic-clamp studies in animal studies, glucose utilization in the medial basal hypothalamus, locus coeruleus, and motor cortex was found to be decreased (despite normal plasma glucose levels) [79]. The decreased glucose utilization in these brain regions induced marked elevations of serum corticosterone levels [79].

Only a few numbers of studies have investigated effects of insulin on the HPA axis in humans. Fruehwald-Schultes et al. reported that in human subjects acute hyperinsulinemia under euglycemic conditions induced an increase in plasma ACTH and cortisol concentrations in a similar way as previously reported in animal studies [80]. Comparable to animal studies, stimulatory effects of insulin on the HPA axis activity were also only found when relatively high (supra-physiological) doses of insulin were administered, but were absent during administration of relatively low insulin doses [80].

**Figure 6:**
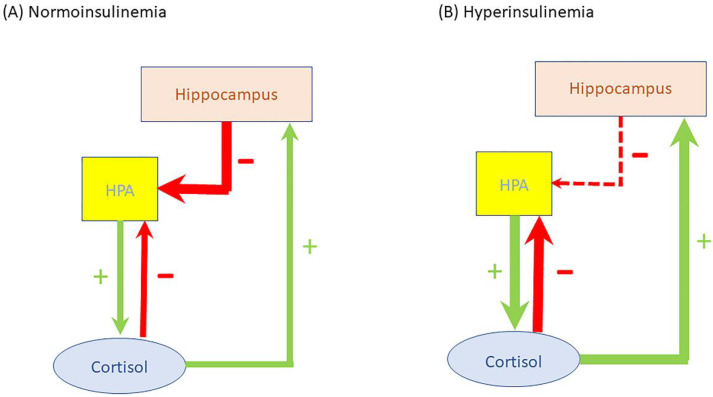

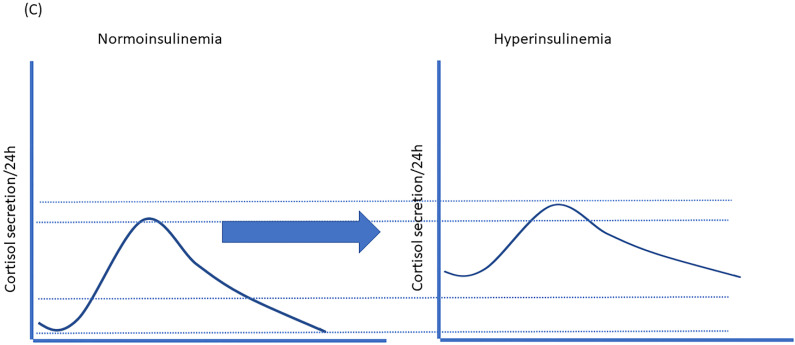
Insulin can cross the blood brain barrier and exert direct effects at the hippocampus. In normoinsulinemic conditions, the hippocampus has inhibitory control over the HPA axis preventing excess cortisol release. Cortisol in turn has negative feedback on the HPA activity and positive feedback on the hippocampus (**A**). In hyperinsulinemic conditions the inhibitory activity of the hippocampus on the HPA axis is decreased (releases “the brake”). Consequently, the homeostatic setpoint (the predetermined level) of the HPA axis activity will change: cortisol secretion per 24 h increases (compared to normoinsulinemic conditions) and thereby induces a state of “functional hypercortisolism” (**B**). The exaggerated cortisol secretion per 24 h during hyperinsulinemia can be explained by increased forward drive to the HPA axis, and/or reduced sensitivity of the HPA axis to negative feedback by cortisol (**C**). Stimulatory effects are shown in green and inhibitory effects are shown in red. See text for more details.

As mentioned previously, circulating insulin can cross the blood brain barrier and thereby exert direct effects at the hippocampus, the hypothalamus, and the pituitary [80]. In normoinsulinemic conditions, the hippocampus inhibits the HPA axis and thereby prevents excess cortisol release [81] (Figure 6A). However, in hyperinsulinemic conditions the inhibitory activity of the hippocampus on the HPA axis is decreased (releases “the brake”). Consequently, the homeostatic setpoint (the predetermined level) of the HPA axis activity is set to a higher level: cortisol secretion per 24 h increases (compared to normoinsulinemic conditions), thereby inducing a state of “functional hypercortisolism” [81,82] (Figure 6B,C). The exaggerated cortisol secretion per 24 h during hyperinsulinemia can be explained by increased forward drive to the HPA axis, and/or reduced sensitivity of the HPA axis to negative feedback by cortisol [16,83,84,85] (Figure 6C).

Both birth and infant weights have been found to be inversely associated with the prevalence of impaired glucose tolerance, the metabolic syndrome, type 2 diabetes, and polycystic ovary syndrome later in life [86]. In addition, it has become clear that intra-uterine environment may also have long-lasting and permanent effects on the activity of the HPA axis [87,88,89]. Urinary excretion of total glucocorticoid metabolites in a population sample of 9-year-old children was higher in children who had a low birthweight [87]. In a study in 60- to 70-year-old men, fasting plasma cortisol concentrations were inversely related to birth weight and body mass index [88]. Furthermore, it was shown that pituitary and adrenal progenitor cells exposed to hyperinsulinemia are metabolically primed to a hyper-functional state of the HPA axis and enhanced cortisol production later in life [89]. This latter mechanism might explain how hyperinsulinemia early in life increases the risk for developing the metabolic syndrome in adulthood [89].

## 11. HPA Activity in Obesity and the Metabolic Syndrome

Earlier studies proved that the total daily cortisol production rate is somewhat enhanced in obesity and the metabolic syndrome [90,91,92,93]. This is further supported by data showing that the HPA axis responsiveness in obesity, particularly when there was visceral obesity, was enhanced to stimuli such as acute stress, hypoglycemia, a standard meal, or CRH/arginine vasopressin [83,91,94]. In addition, ACTH hyper-responsiveness after CRH/AVP stimulation also appeared to be related to hyperinsulinemia, although the exact underlying mechanism(s) of this relationship is unclear [94].

Increased adipose tissue 11b-HSD-1 expression and activity is also found in human obesity [83]. In subjects with (central) obesity, contrary to those with Cushing syndrome, basal plasma cortisol levels are often not elevated [83]. Despite hyperinsulinemia-mediated increase in cortisol production in overweight and obese subjects, plasma cortisol levels are either similar to or lower than those in non-obese individuals, suggesting that there is also an increased plasma cortisol clearance [95,96]. Insulin-mediated changes in cortisol binding globulin (CBG) concentrations may play a role in the increased plasma cortisol clearance [95,96]. It has been found that plasma CBG levels were negatively related with the insulin response to a glucose load and this relationship was independent of insulin sensitivity [97]. Hyperinsulinemia might decrease CBG liver production and/or increase CBG plasma clearance rate or may have both effects [97]. A decrease in CBG plasma levels may increase plasma cortisol clearance and act to amplify the availability of metabolically free cortisol at peripheral target organs at the tissue level [98]. The reduction in plasma levels of cortisol caused by CBG might decrease feedback inhibition on the hypothalamus and/or pituitary. This in turn would cause upregulation of the HPA axis with increased cortisol synthesis in the adrenals. Thus hyperinsulinemia-induced decrease in plasma CBG levels may help to explain why in subjects with hyperinsulinemia and (central) obesity, lowered plasma total cortisol levels are found despite the presence of an increased HPA activity and an increased total daily cortisol production [95]. However, there are likely to be several other mechanisms involved in the increased HPA activity, such as the negative feedback effects of cortisol on the HPA axis and the tissue selective pleotropic effects of hyperinsulinemia (see below).

## 12. Direct Effects of Insulin on the Adrenals

Penhoat and colleagues showed insulin receptors in bovine adrenal fasciculata cells [99]. Furthermore, they demonstrated in a series of in vitro experiments that insulin enhances the steroidogenic and cAMP response to ACTH [99]. The enhanced steroidogenic responsiveness of insulin-treated cells was related to an enhanced capacity to produce pregnenolone and an increased activity of several steroid hydroxylases [99]. Thus, by increasing local sensitivity of the adrenals to ACTH, insulin may contribute in the adrenals to enhanced adrenocorticotropic effects (Figure 5). It has been further postulated that insulin may directly influence adrenal steroidogenesis independent of ACTH actions. It was demonstrated that insulin regulates steroidogenesis in the adrenals by activating steroidogenic factor 1 (SF-1) and its steroidogenic target genes important for steroid hormone synthesis through a mechanism independent of the canonical CRH/ACTH/MC2R/PKA pathway and its adrenocorticotropic effects [100] (Figure 5). Thus, insulin may be directly involved, at least in part, in increased cortisol production rate by the adrenal glands (see also below, paragraph *Effects of insulin on local cortisol metabolism*).

Cross-sectional studies suggest a significant association between hyperinsulinemia and the rising number of adrenal incidentalomas/tumors [101]. Hyperinsulinemia may be responsible for adrenal cellular proliferation and tissue proliferation and exerts its mitogenic effects directly by acting on the insulin receptors or, indirectly, by reducing the production of IGFBP-1 in the liver and thereby increasing the bioavailability of free IGF-I [102] (Figure 5). Interestingly, adrenal gland volume has been suggested as a marker of HPA axis activity reflecting chronic cortisol burden and it was recently reported in a population-based cohort study that mean adrenal gland volume measured by MRI was significantly greater in subjects with prediabetes and diabetes than in healthy controls [103,104].

## 13. Effects of Insulin on Local Cortisol Metabolism

Cortisol metabolism is not only centrally regulated. As discussed previously, in target tissues local cortisol concentrations are tissue-specific controlled at the prereceptor level through 11b-HSD1, which interconverts hormonally inactive cortisone to active cortisol. In this way local tissue cortisol concentrations are regulated independent of circulating cortisol concentrations [105]. Insulin is probably one of the main factors that exert effects on local cortisol metabolism at the prereceptor level by dynamically regulating 11β-HSD1 [106,107]. Data from transgenic mice selectively over-expressing 11βHSD-1 in adipocytes provide evidence for a causal role of 11βHSD-1 in the development of visceral obesity and the metabolic syndrome [108]. Conversely, 11βHSD-1 knockout mice have lower glucocorticoid levels in adipose tissue and are protected from dietary-induced obesity and show an improved lipid profile, hepatic insulin sensitization, and a potentially atheroprotective phenotype [109].

Through a posttranscriptional mechanism that involves activation of the p38 MAPK signaling pathway, insulin stimulates adipocyte 11β-HSD1 activity and expression in concentration-dependent manner [107]. Hyperinsulinemia-induced expression of 11β-HSD1 may stimulate local cortisol secretion in adipocytes through enhanced conversion of cortisone to cortisol and in this way may contribute to increased cortisol concentrations in vivo [110] (Figure 5). The increased cortisol secretion will stimulate glucocorticoid receptor activation and promote central adiposity [106,107,111,112].

In contrast, in the liver, hyperinsulinemia suppresses 11β-HSD1 expression and activity, leading to a decreased hepatic conversion of cortisone into cortisol [111]. It has been hypothesized that decreased hepatic conversion of cortisone into cortisol due to hyperinsulinemia-suppressed 11β-HSD1 activity in the liver represents a physiological mechanism to improve hepatic insulin sensitivity and to lower fasting plasma glucose levels [111].

## 14. Can Hyperinsulinemia-Induced Activation of the HPA Axis Be Modified?

A number of different treatment approaches can prevent, reduce, and reverse hyperinsulinemia and insulin resistance, obesity, and metabolic syndrome, regardless of whether hyperinsulinemia or insulin resistance “comes first” (i.e., calorie restricted, low carbohydrate diets and gastric bypass surgery). What needs to be further investigated is the effect of these interventions on the HPA axis and “functional hypercortisolism” hypothesis as outlined in this manuscript. This would help elucidate the role of hyperinsulinemia and cortisol in the pathophysiology of the metabolic syndrome.

At this moment it is unclear whether there is a successful strategy to modify hyperinsulinemia-induced “functional hypercortisolism” in subjects prone to develop the metabolic syndrome. Most studies in humans have not shown consistent effects of energy restriction on activation of the HPA axis. It has been reported that long-term moderate energy restriction in (obese) humans resulted in increases, decreases, as well as no change in HPA axis function [113,114,115,116,117,118].

Sodium–glucose cotransporter 2 (SGLT2) inhibitors may also indirectly reduce hyperinsulinemia [12]. Sodium–glucose cotransporter-2 inhibition by SGLT2 inhibitors leads to glycosuria and the lowering of plasma glucose [119]. Another consequence of using SGLT2 inhibitors is the development of a relative hypoinsulinemia, which is part of the first line of defense against hypoglycemia [120]. Of note in this context are findings of a beneficial effects of sodium–glucose transport protein 2 (SGLT-2) inhibitors on central nervous (hypothalamic?) actions [121]. It was recently reported that the SGLT-2 inhibitor tofogliflozin decreased serum ACTH and cortisol levels in subjects with type 2 diabetes, indicating that tofogliflozin also influences the HPA axis [122]. Moreover, metformin, an insulin-sensitizer used as the first-line drug for treating type 2 diabetes, does not only lower plasma insulin and glucose levels, but also can reduce ACTH levels and cortisol [123,124]. For a long time, it has been unclear how the glucose-lowering effect of metformin was related to AMPK activation. Recently, it was found that metformin in rat pituitary cells induced AMPK/liver X receptor a (LXRa) phosphorylation which was followed by pro-opiomelanocortin (POMC) suppression [124]. The authors suggested that part of the anti-hyperglycemic effect of metformin could be attributed to reduced POMC/adrenocorticotropic hormone (ACTH)/cortisol levels following AMPK phosphorylation in the pituitary gland [124]. However, whether reduced activity of the HPA axis was secondary to metformin-mediated effects on hyperinsulinemia was not investigated in this study.

To date, there is no evidence that normalizing hyperinsulinemia in subjects with the metabolic syndrome can reduce (increased) activation of the HPA axis. It is also unknown whether this strategy would exert long-term beneficial effects on health. Therefore additional (longitudinal) studies are required to determine the importance of chronic hyperinsulinemia as driving force for increased HPA axis activity in subjects with the metabolic syndrome. Moreover, when these studies provide evidence for hyperinsulinemia as driving force for increased HPA axis activity in subjects with the metabolic syndrome, future research should focus on developing (new) strategies/drugs that can successfully prevent/reduce hyperinsulinemia-induced “functional hypercortisolism” in subjects with the metabolic syndrome.

In conclusion, emerging data suggest that chromic hyperinsulinemia is the driving force for increased HPA axis activity in subjects with the metabolic syndrome. Despite an increased HPA activity, plasma cortisol levels in subjects with the metabolic syndrome are often not elevated, due an increased plasma cortisol clearance. Nevertheless, subjects with the metabolic syndrome show a state of “functional hypercortisolism”. This “functional hypercortisolism” by antagonizing insulin actions may prevent hypoglycemia, disturb energy homeostasis, and shift energy fluxes away from muscle toward fat stores. Synergistic effects of hyperinsulinemia and “functional hypercortisolism” promote fat accumulation in visceral fat cells and so contribute to abdominal obesity. Chronic hyperinsulinemia-induced activation of the HPA axis may play an important etiological role in the development of the metabolic syndrome and all its consequences.

## Figures and Tables

**Table 1:** Overlap and differences in clinical characteristics between the metabolic syndrome and Cushing syndrome.

	Metabolic Syndrome	Cushing Syndrome
Plasma Insulin levels	↑↑	↑ * or ↓ **
insulin resistance	+	+
AbdominalObesity	+	+
impaired glucose tolerance	+	+
Hypertriglyceridemia	+	+
Hypertension	+	+
HPA Activity	↑	↑
Plasma Cortisol	↓/N	↑
24 h-Free Cortisoluria	↑	↑

+ = present; ↑ or ↑↑ = elevated; N = normal; ↓ = decreased; * “relative hypoinsulinemia”: insulin levels are increased but lower than would be expected for the actual level of glucose; ** about 20% of patients with Cushing syndrome will finally develop frank diabetes.

## Data Availability

Not applicable.
